# Calpain-1 weakens the nuclear envelope and promotes the release of neutrophil extracellular traps

**DOI:** 10.1186/s12964-024-01785-6

**Published:** 2024-09-09

**Authors:** Jeeshan Singh, Leticija Zlatar, Marco Muñoz-Becerra, Günter Lochnit, Irmgard Herrmann, Felix Pfister, Christina Janko, Jasmin Knopf, Moritz Leppkes, Janina Schoen, Luis E. Muñoz, Georg Schett, Martin Herrmann, Christine Schauer, Aparna Mahajan

**Affiliations:** 1 Department of Medicine 3 - Rheumatology and Immunology, Friedrich-Alexander-Universität Erlangen-Nürnberg and Uniklinikum Erlangen, Erlangen, Germany; 2 Deutsches Zentrum Für Immuntherapie (DZI), Friedrich-Alexander-Universität Erlangen-Nürnberg and Uniklinikum Erlangen, Erlangen, Germany; 3 Protein Analytics, Institute of Biochemistry, Faculty of Medicine, Justus-Liebig-University Giessen, Giessen, Germany; 4 Department of Otorhinolaryngology, Head and Neck Surgery, Section of Experimental Oncology and Nanomedicine (SEON), Uniklinikum Erlangen, Erlangen, Germany; 5 Department of Pediatric Surgery, University Medical Center Mannheim, University of Heidelberg, Mannheim, Germany; 6 Department of Medicine 1 - Gastroenterology, Pneumology and Endocrinology, Friedrich-Alexander-Universität Erlangen-Nürnberg and Uniklinikum Erlangen, Erlangen, Germany

**Keywords:** Nesprin-1, Calpain-1, Neutrophil extracellular traps (NETs), Nuclear envelope break down

## Abstract

The inducers of neutrophil extracellular trap (NET) formation are heterogeneous and consequently, there is no specific pathway or signature molecule indispensable for NET formation. But certain events such as histone modification, chromatin decondensation, nuclear envelope breakdown, and NET release are ubiquitous. During NET formation, neutrophils drastically rearrange their cytoplasmic, granular and nuclear content. Yet, the exact mechanism for decoding each step during NET formation still remains elusive. Here, we investigated the mechanism of nuclear envelope breakdown during NET formation. Immunofluorescence microscopic evaluation revealed a gradual disintegration of outer nuclear membrane protein nesprin-1 and alterations in nuclear morphology during NET formation. MALDI-TOF analysis of NETs that had been generated by various inducers detected the accumulation of nesprin-1 fragments. This suggests that nesprin-1 degradation occurs before NET release. In the presence of a calpain-1, inhibitor nesprin-1 degradation was decreased in calcium driven NET formation. Microscopic evaluation confirmed that the disintegration of the lamin B receptor (LBR) and the collapse of the actin cytoskeleton occurs in early and later phases of NET release, respectively. We conclude that the calpain-1 degrades nesprin-1, orchestrates the weakening of the nuclear membrane, contributes to LBR disintegration, and promoting DNA release and finally, NETs formation.

**Supplementary Information:**

The online version contains supplementary material available at 10.1186/s12964-024-01785-6.

## Introduction

Neutrophils are the most abundant white blood cells and also the first immune cells that are recruited at the site of infection and inflammation. After infiltration, neutrophils render different effector functions, such as phagocytosis and degranulation. A further effector function of neutrophils involves the release of decondensed chromatin, decorated with cytoplasmic and granular proteins [1]. The central function is to trap and degrade pathogens, foreign matter, cytokines and chemokines extracellularly inside a meshwork formed by decondensed DNA [2, 3]. This structure is referred to as neutrophil extracellular traps (NET), and the process is known as NET formation. It involves extensive nuclear and cytoplasmic remodelling [1]. It can be categorized as follows: neutrophil stimulation, chromatin decondensation, nuclear envelope breakdown, complexing of chromatin with granular and cytoplasmic proteins, and release of this complexed decorated chromatin into the extracellular space [1, 2]. The molecular, biochemical and structural mechanisms supporting nuclear envelope breakdown during NET formation are still elusive.

A wide range of stimuli can induce NET formation. Phorbol 12-myristate 13-acetate (PMA) is potent NET inducer. PMA induced NET formation involves protein kinase C (PKC), calcium influx, Nicotinamide adenine dinucleotide phosphate (NADPH) oxidase, myeloperoxidase (MPO), neutrophil elastase (NE) [4]. While PMA is not physiologically relevant NET inducer. Ionomycin, A23187 calcium ionophore and bacterial toxin-nigericin (potassium ionophore) induced NET formation is calcium dependent and involves PAD4 mediated DNA citrullination and chromatin decondensation [5]. Several studies showed that the genetic or pharmacological inhibition of PAD4 resulted in reduced or impaired release of NETs [6–8]. Hence, in this study we have used NET inducers like calcium ionophore A23187 and bacterial toxin-nigericin to investigate the molecular mechanism of calcium dependent NET formation [9].

The condensed chromatin in a neutrophil is encased within the nuclear envelope, which safeguards and segregates the chromatin material and nucleoplasm from cytoplasm. It is a double lipid membrane composed of an outer (ONM) and an inner nuclear membrane (INM). The perinuclear space consists of the linker of nucleoskeleton and cytoskeleton (LINC) complex consisting of SUN protein family members and multiple isoforms of nesprin [10]. Nuclear envelope spectrin-repeat proteins (Nesprins) exist in four isoforms, namely nesprin1-4 [10–12]. The nesprin-1 isoform contains an N-terminal actin-binding domain, a central rod consisting of several spectrin repeats and a C-terminal transmembrane domain [10, 12]. The giant isoform nesprin-1 forms an indispensable bridge between the cytoplasmic cytoskeletal protein actin and the INM protein lamin B receptor (LBR). This underlines the importance of nesprin-1 in nuclear anchorage and positioning [13].

Calpain is a calcium-dependent intracellular cysteine protease and occurs in several isoforms. Calpain -1 and -2 are present in neutrophils [14]. Calpain cleaves a wide variety of protein substrates involved in calcium signaling during chemotaxis, cell division, granule secretion and apoptosis [15, 16]. The substrates for calpain are mainly cytoskeleton proteins [17]. Purified calpains can proteolyze several high molecular weight nuclear proteins at physiological calcium contractions [18]. It typically targets inter-domain regions. This suggests that calpain plays an essential physiological role in the turnover of nuclear proteins. It has been shown that calpain and calpain inhibitors are critical for neutrophil chemotaxis [19]. Calpain-1 together with peptidyl arginine deiminase-4 (PAD–4) in neutrophils cause chromatin decondensation during the process of NET formation [20].

We conclude that calpain-1 degrades the nuclear envelope protein nesprin-1 during calcium-dependent neutrophil activation. This weakening of the nuclear envelope mechanistically promotes chromatin release during NET formation.

## Material and methods

### Ethics statement

All individuals included in this study provided written and informed consent. All experiments were performed using human material according to the 1964 Helsinki Declaration and its later amendments or comparable ethical standards for research involving human subjects (permit # 243_15B). In addition, institutional approval was obtained from each local Ethical Committee.

### Isolation of human neutrophils

Whole blood from healthy human donors was freshly drawn into EDTA tubes (S-Monovette® EDTA K3E, 9 ml, Sarstedt). We began the isolation of healthy neutrophils by density gradient centrifugation at 350 g for 30 min at room temperature with histopaque (Histopaque®-1077, Sigma-Aldrich). The high-density layer resting on the red blood cell layer was subjected to hypotonic erythrocyte lysis. We then resuspended the neutrophil pellet in Dulbecco´s Phosphate Buffered Saline (DPBS, ThermoFisher Scientific) and determined the viable cell concentration by staining with acridine orange/propidium iodide (Logos Biosystems) using the Luna-FL™ Dual Fluorescence Cell Counter (Logos Biosystems).

### NETs formation assay

Neutrophils were adjusted to a concentration of 6 million cells/ml with DPBS and seeded (~ 150.000 cells/well) in RPMI-1640 (Roswell Park Memorial Institute, Gibco) medium in a 96-well plate (Greiner, flat bottom transparent polystyrene plate). SYTOX™ Green, a membrane-impermeable DNA dye 2.5 μM (S7020, Thermo Fisher Scientific), calcium (1 mM) (Calcium Chloride, Art No. CN93.1, Roth) or ethylene glycol-bis (β-aminoethyl ether)-N,N,N′,N′-tetraacetic acid (EGTA) 1 mM (E0396-10G, Sigma-Aldrich) were added to the RPMI medium before addition of the NETs stimuli. Neutrophils were incubated with either 10 µM calcium ionophore A23187 (Sigma Aldrich), 5 µg/ml ionomycin (Sigma Aldrich), 3 µg/ml LPS from Klebsiella pneumoniae (Sigma), 15 µM nigericin or 10 ng/ml PMA (Sigma Aldrich) at 37 °C and 5% CO_2_ for 4 h. For the inhibition assay, neutrophils were pre-incubated with 100 µM calpeptin (Sigma Aldrich) at 37 °C and 5% CO_2_ for 30 min before stimulation.

### Quantification of NETs

The SYTOX™ Green DNA stain was utilized to quantify the DNA externalization by neutrophils during the formation of NETs. The increase in the SYTOX™ Green fluorescence intensity, at an excitation wavelength of 485 nm and emission wavelength of 535 nm, was measured for 4 h at 37 °C and 5% CO_2_ at the plate reader Infinite® F200 Pro fluorometer (TECAN, Crailsheim, Germany). Relative fluorescence units (RFU) were determined as the ratio of the fluorescence at the indicated time point and time point 0 min and multiplied by 100. The formation of NETs after 4 h was fixed with 1% paraformaldehyde (PFA) and examined using BZ-X710 fluorescence microscope (Keyence, Neu-Isenburg, Germany).

### Neutrophil nuclei isolation

Human blood neutrophils were isolated as described earlier. Further, neutrophil nuclei were isolated via a biochemical fractation protocol described previously [21]. Nuclei pellets were suspended in buffer (100 mM Tris pH7,6, 10 mM CaCl_2_, 5 mM DTT) containing SYTOX™ Green and incubated for 1 h. The following components 10 mM CaCl_2_, 5 µg/ml PAD4 (Cayman), and/or 15 µg/ml calpain-1 (Sigma-Aldrich) were added as indicated in the experiment. Decondensation of nuclei was observed using BZ-X710 fluorescence microscope (Keyence).

### Immunocytochemistry

Freshly isolated neutrophils were adjusted to a concentration of 6 million cells/ml in PBS. Twenty-five μl of this suspension (~ 150.000 cells) were seeded in 8-well chamber slides (Cat no. 177445, Nunc™ Lab-Tek™ Chamber Slide System, Thermo Fisher Scientific) also containing 155 μl of RPMI (Roswell Park Memorial Institute) 1640 medium with 1 mM calcium (Sigma-Aldrich). Neutrophils were stimulated with 10 µM calcium ionophore A23187 (Sigma-Aldrich), 5 µg/ml ionomycin (Sigma-Aldrich) or 10 ng/ml PMA (Sigma-Aldrich) at 37 °C and 5% CO_2_. The formation of NETs was halted at different time points by fixation with 2% PFA for 30 min at room temperature, followed by washing with PBS twice. Samples were permeabilized with 0.1% Triton X-100 in PBS for 6 min at RT and washed thrice with PBS. Samples were blocked with blocking buffer (10% fetal calf serum FCS or 10% normal goat serum NGS + 2% bovine serum albumin BSA + 0.1% Triton X-100 + 0.05% Tween-20 in PBS) for one hour at room temperature (RT). Unconjugated primary antibody for nesprin-1 (1:50, PA5-115640, Invitrogen) and/or directly conjugated anti-LBR (1:50, AF488-anti-LBR, ab201532) were diluted in blocking buffer and incubated overnight at 4ºC. Samples were washed three times with PBS. Secondary antibody anti-rabbit IgG cross-adsorbed Alexa Fluor 647 antibody (1:400, A-31573, Molecular Probes) and DNA staining dye 4′,6′-diamidino-2phenylindole DAPI (1:1000 from 1 mg/ml stock) were added and incubated on the cells for 1.5 h at RT in the dark. Slides containing the cells were rinsed two times with PBS and one time with distilled water. Negative controls were prepared simultaneously, lacking the primary antibody for each staining. Samples were then embedded in DAKO fluorescent mounting medium (Agilent Technologies, Santa Clara). Samples were observed using a BZ-X710 fluorescence microscope (Keyence).

### F-actin immunofluorescence microscopy

Neutrophils and stimulated neutrophils were fixed with 2% PFA for 30 min at room temperature, followed by washing with PBS twice. Samples were permeabilized with 0.5% Triton X-100 in PBS for 6 min at RT and washed thrice with PBS. This was followed by the staining for nesprin-1 and DNA dye DAPI as described in section immunocytochemistry. Then samples were washed with PBS and stained with 5 µM SiR actin-Cy5 in PBS for 2 h (SiR-actin kit, Spirochrome). Samples were washed with PBS twice and one time with distilled water. Negative controls were prepared simultaneously, lacking the primary antibody for each staining. Cells were then embedded in DAKO fluorescent mounting medium (Agilent Technologies, Santa Clara). Samples were observed using BZ-X710 fluorescence microscope (Keyence).

### FIt-SNE plot formation and data analysis

Microscopy images of regions of interest were processed in Photoshop CC 2018 (Adobe, Munich, Germany). The morphometric values of circularity, area, time of incubation, grey value of DAPI, and Nesprin of single cells were recorded and exported into an R data table object. In order to simplify the visualization of the morphometric data, dimensionality reduction using Fit-SNE was carried out through the ‘*run.fitsne()*’ function and FIt-SNE plots were created with the function ‘*make.colour.plot()*’ available in the Spectre R package with detailed instructions and source code accessible https://github.com/ImmuneDynamics/spectre [22].

### Protein separation and MALDI-TOF MS spectrometry

Isolated human blood neutrophils were adjusted to a concentration of 20 million cells/ml of Hank’s buffered saline solution (HBSS) containing calcium and magnesium in Eppendorf tube. Neutrophils were stimulated with 10 ng/ml PMA (Sigma), 300 pg/cell monosodium urate (MSU) crystals, 72 mM sodium bicarbonate or 1 mg/ml diamond D10 nm (Sigma-Aldrich) at 37˚C and 5% CO_2_. After 4 h of incubation, samples were centrifuged at 1000 rpm for 5 min. Lysates of cell pellets were prepared in Laemmli buffer at 95 °C for 15 min. Further proteins were separated using 10% Tris-Tricine gel electrophoresis. Tris-Tricine gel was prepared as described as [23]. This gel was assessed via silver staining using a ProteoSilver Plus silver stain kit (Sigma-Aldrich). Silver staining of gels was performed according to the manufacturer's instructions. The gels were fixed overnight at 4 °C. After developing for 3 to 5 min, the gel was re-equilibrated in water until the bands were excised for mass spectrometry (MS).

Tryptic digestion of proteins: Excised bands were digested after reduction and carbamidomethylation with trypsin. Tryptic peptides were eluted from the gel plugs with 1% trifluoric acid.

Matrix-assisted laser-desorption ionization time-of-flight mass spectrometry (MALDI-TOF–MS): MALDI-TOF–MS was performed on an Ultraflex TOF/TOF mass spectrometer (Bruker Daltonics, Bremen) equipped with a nitrogen laser and a LIFT-MS/MS facility. The instrument was operated in the positive-ion reflectron mode using 2.5-dihydroxybenzoic acid and methylendiphosphonic acid as matrix. Sum spectra consisting of 200–400 single spectra were acquired. For data processing and instrument control the Compass 1.4 software package consisting of FlexControl 4.4, FlexAnalysis 3.4 4, Sequence Editor and BioTools 3.2 and ProteinScape 3.1. were used. External calibration was performed with a peptide standard (Bruker Daltonics).

Data acquisition and analysis: Proteins were identified by MASCOT peptide mass fingerprint search (http://www.matrixscience.com) using the Uniprot Human database (version 20,151,209, 92,013 sequence entries; 56,594,998 residues; *p* < 0.05). For the search, a mass tolerance of 75 ppm was allowed and oxidation of methionine as variable modification and carbamidomethylation of cysteine as fixed modification were used.

### Live cell imaging

Freshly isolated human blood PMNs were adjusted to a concentration of 1 million cells/ml in PBS. Neutrophils were then added to 800 μl of RPMI (Roswell Park Memorial Institute) 1640 medium containing 1 mM calcium, with or without 100 µM calpeptin, and 10 µM of A23187 as NET inducer. Neutrophils were pre-incubated with 100 µM of calpeptin for 30 min at 37 °C and 5% CO_2_. The assay was performed in a µ-dish (35 mm, high ibidi plate, Part of 81,156, ibidi GmbH) and was visualized via 3D-Cell Explorer Nanolive microscope (Nanolive SA, Switzerland) measuring real-time kinetics of NETs formation for 4 h where each frame was acquired every 30 s.

### Statistical analyses

Statistical analyses were performed in Excel 2019 (Microsoft) or GraphPad Prism 9. For comparison between two groups, unpaired t-test was performed according to the distribution of data. For multiple variables comparison, Tukey’s multiple comparison test, two-way ANOVA and Bonferroni’s multiple comparison tests were performed as indicated in the figure legends.

## Results

### Extracellular Ca^2+^ activates PAD-4 and calpain-1 to promote NET formation.

**Fig. 1:**
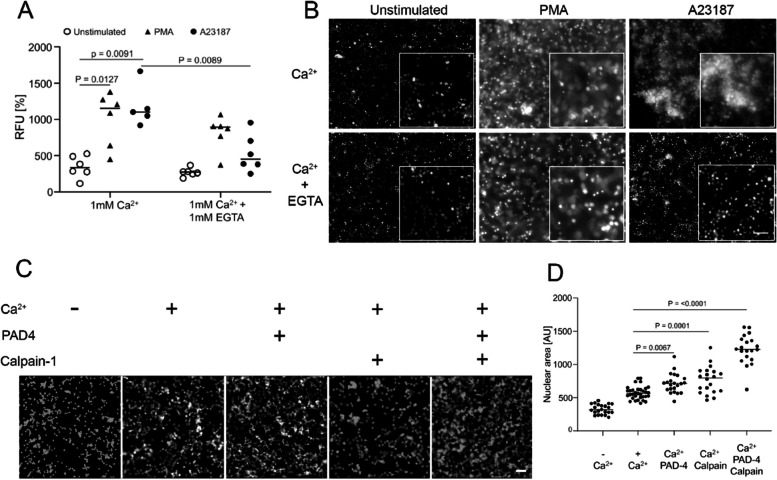
Ca^2+^ Influx activates PAD-4 and calpain-1 and promote neutrophil extracellular trap (NET) formation. **A** shows chromatin externalization from healthy neutrophils (n > 5) treated with PBS unstimulated (○), 100 ng/ml phorbol 12-myristate 13-acetate (PMA ▲), or 10 µM calcium ionophore A23187 (●) in the presence and the absence of calcium chelator EGTA. Data were analyzed using Tukey's multiple comparisons tests. Note, the calcium-driven NET formation was abrogated by EGTA. **B** Microscopic analyses of SYTOX Green stained DNA from neutrophils and NETs at 4 h after stimulation. **C** Microscopic examination of the swelling of isolated nuclei from neutrophils in the presence and/or absence of PAD4, calpain and calcium. **D** Morphometry of the nuclear sizes from (**C**). Note that calcium, PAD4, and calpain-1 act synergistically in decondensation/swelling of nuclei. Tukey's multiple comparisons test was used to evaluate the differences among means. The scale bars are 100 and 50 µm in (B) and (**C**), respectively

To determine the function of Ca^2+^ in NET formation, we isolated neutrophils from healthy donors and stimulated them with PMA or the Ca^2+^ ionophore A23187. This was followed by the plate reader-based quantification of NET formation in which the externalization of DNA was measured in terms of the SYTOX™ Green fluorescence signal [2, 24]. In the presence of the cell impermeant calcium chelator ethylene glycol-bis (β-aminoethyl ether)-N, N, N′, N′-tetraacetic acid (EGTA), we found reduced NET formation especially for A23187-induced NETs (Fig. 1A). Microscopic evaluation of chromatin externalization from A23187 stimulated neutrophils showed less DNA decondensation in the presence of EGTA (Fig. 1B). Elevated cytosolic calcium activates certain enzymes such as peptidyl arginine deiminase-4 (PAD-4) and calpain-1. This resulted in nuclear decondensation, a hallmark of NET formation. This was further confirmed when isolated neutrophil nuclei, incubated with recombinant PAD-4, calpain-1 and calcium showed increased nuclear swelling (Fig. 1C). Morphometry of nuclei from Fig. 1C revealed significantly more nuclear swelling in the presence of calcium, PAD-4, and calpain (Fig. 1D). Here we demonstrate that a concerted action of Ca^2+^, PAD-4 and calpain sensitizes neutrophils for chromatin decondensation, nuclear swelling, and formation of NET in calcium ionophore stimulated neutrophils [20].

### Nesprin-1 protein maintains the nuclear structure of neutrophils

**Fig. 2:**
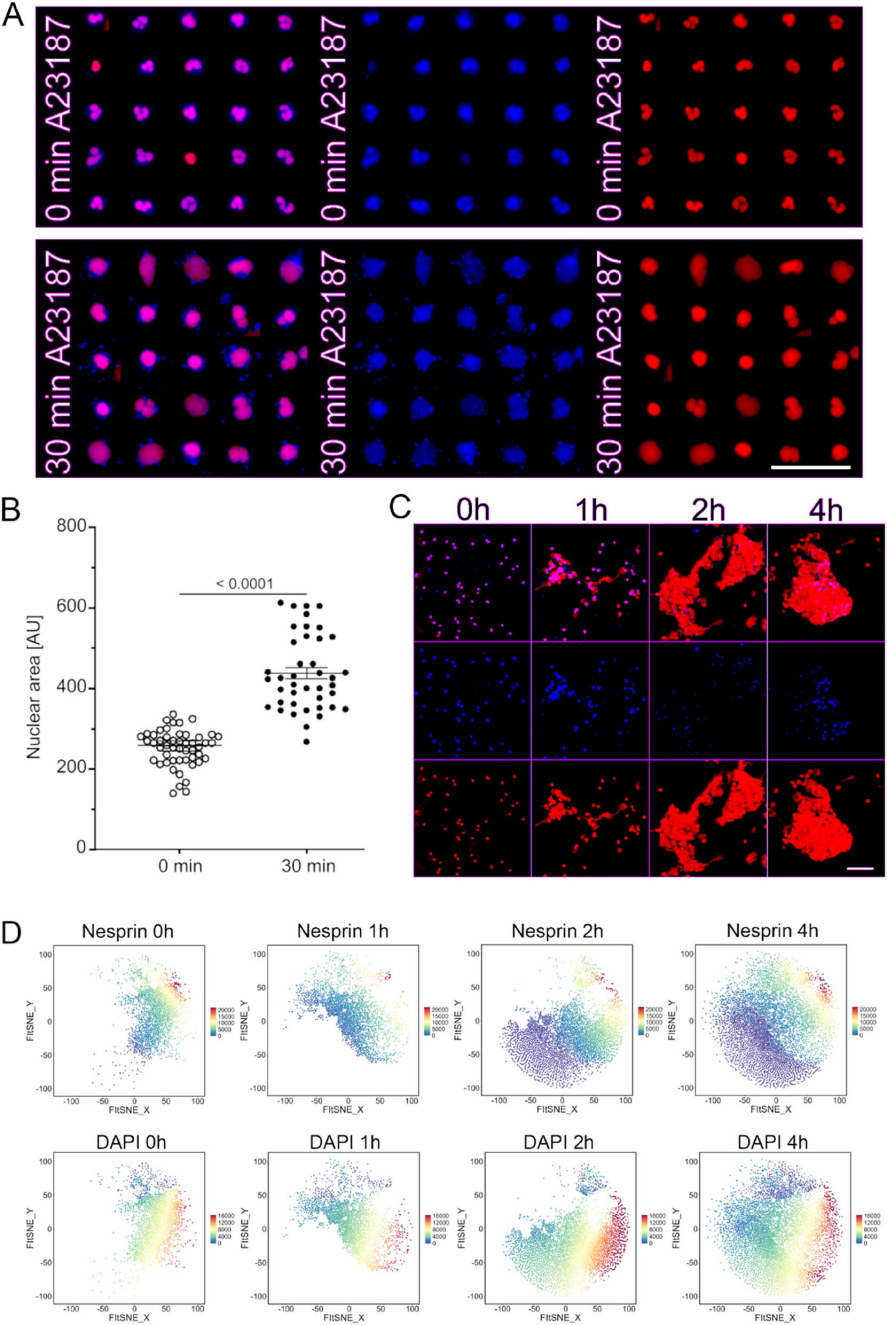
Degradation of nesprin-1 in neutrophils precedes nuclear envelope rupture and chromatin externalization. **A** Immunofluorescence staining of neutrophils for DNA (DAPI displayed in red) and nesprin-1 (displayed in blue) before and 30 min after stimulation with calcium ionophore A23187 (1 mM Ca^2+^). Merge composites (left), nesprin-1 (middle), and DNA (right) staining demonstrates the breakdown of nesprin-1 from neutrophil nuclear membrane within 30 min. It leaves dot-like nesprin-1 remnants, and is accompanied by the loss of nuclear lobes. Scale bar is 50 µm. **B** Quantitative evaluation of the nuclear site 30 min after stimulation with A23187 shows nuclear swelling. Means were analyzed by Unpaired t-test. **C** A kinetic composite displaying the overall degradation of nesprin-1 and formation of NETs over 4 h after stimulation with calcium ionophore A23187 (1 mM Ca^2+^). **D** FIt-SNE plots displaying the fluorescence intensity for nesprin-1 and DNA staining (DAPI) of ionophore A23187-stimulated neutrophils

To investigate the connection of Ca^2+^ in the nuclear envelope breakdown during NET formation, we stimulated neutrophils with A23187 in the presence of 1 mM extracellular Ca^2+^. Cells were immunostained for nesprin-1. The chromatin was counterstained with DAPI. The NET formation was monitored for 4 h. Induction of NET formation with A23187/Ca^2+^ resulted in the distortion of the nuclear lobes within 30 min and nuclear swelling. The remains of nesprin-1 in neutrophils were scattered as dot-like flecks (Fig. 2A & S7) after 30 min of incubation with A23187. We observed robust nuclear swelling (Fig. 2B). Next, we examined the fate of nesprin-1 protein at later time points of NET formation. We visualized the kinetics of this process and observed that nesprin-1 underwent further breakdown in a time-dependent manner. With the progression of nesprin-1 deterioration, nuclei collapsed and NETs were generated. In addition, the dot-like structures also decreased (Fig. 2C). After dimensionality reduction using FIt-SNE of nesprin-1 showed the loss of nesprin-1 in various agonist induced NETs (Fig. S1), especially by time point of 1 h with calcium ionophore A23187 (Fig.S2). We also plotted the overall fluorescence intensity of nesprin-1 and DAPI over time (Fig. 2D) as well as by further NET inducers (Fig. S3). Clusters with low nesprin-1signal (violet dots) begin to appear after 1 h as neutrophils initiate the process of NET formation (Fig. 2D). This was further increased at time point 2 h and 4 h suggesting nesprin degradation occurs during NET formation. Clusters with high DAPI signal (yellow and red dots) were seen after 2 h of neutrophil stimulation with NETs inducer (Fig. 2D). This analyses support that during the process of NET formation, the earlier event of nesprin degradation is followed by externalization of decondensed chromatin. Together, these findings indicate the importance of nesprin-1 in the maintenance of the nuclear membrane integrity. The expelling of decondensed chromatin during NET formation is consecutively promoting nuclear breakdown and NET formation.

### Before NET release full-length nesprin-1 is fragmented

**Fig. 3:**
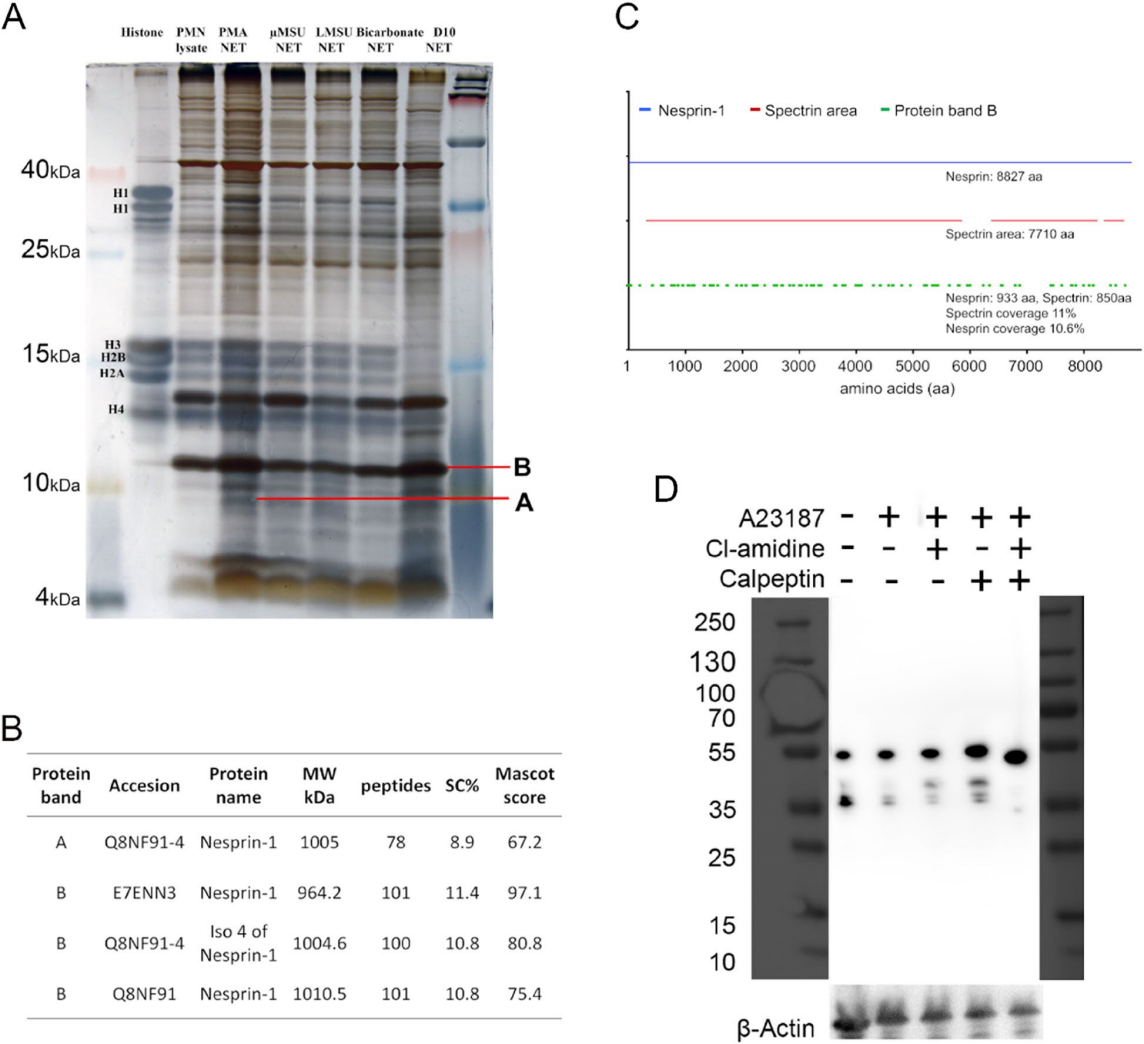
Nesprin-1 is fragmented in the course of NET formation induced by various NETs inducers. **A** Separation of proteins from neutrophils and different NETs preparations using 15% Tris-tricine gel electrophoresis. Protein bands depicted as A and B were identified using MALDI-TOF MS analyses. **B** MALDI-TOF MS analyses of protein bands **A** and **B**. Both protein bands were identified as nesprin-1 protein fragments. MW (molecular weight), SC% (sequence coverage) (**C**) Diagrammatic illustration of sequence analysis of protein band B with nesprin-1 and it’s spectrin repeat area. **D** Western blot analysis shows that the degradation of nesprin was inhibited in the presence of PAD4 inhibitor (Cl-amidine) and calpain inhibitor (Calpeptin) after stimulation of neutrophils with A23184

To understand the process of NET release, we analysed the proteome of NET preparations. Neutrophils isolated from healthy donors were exposed to PMA, monosodium urate (MSU) crystals, alkaline pH (bicarbonate) and nanodiamonds 10 nm (d10). This was followed by electrophoresis and mass spectrometry. We found that proteins with 9—11 kDa contained a plethora of nesprin-1 peptides in various NET preparations (Fig. 3A), as indicated by the information of protein bands in Fig. 3B. These cumulatively covered 87% of the full-length nesprin with 800 and up to 1000 kDa [10] (Fig. 3C). The occurrence of the similar-sized fragments with 10/11 kDa can best be explained by the diagrammatic representation of full of nesprin-1 isoform, the spectrin repeats that stretch across the length of nesprin-1 and the peptides detected from mass spectrometry (Fig. 3C). This suggest the action of a protease that cleaves in the inter-domain regions of the full-length nesprin, as the molecular weight of a single spectrin repeat with 90–110 amino acids which is roughly 11 kDa; as nesprin contain up to 74 spectrin domains oriented like pearls on a string. We hypothesize that the proteolytic shattering of the nesprin-1 weakens the nuclear envelope which breaks and finally, allows NET release.

Furthermore, we performed the western blot analysis of neutrophil lysates using antibody against Nesprin-1. We found protein bands with molecular weight less than 55 kDa in A23187 stimulated neutrophil lysate (Fig. 3D). While, the absence of protein bands less than 55 kDa in A23187 stimulated neutrophils lysate treated with PAD4 and calpain inhibitors suggesting that inhibition of Pad4 and calpain prevents nesprin degradation. This also confirms that pad4 and calpain play a role in degradation of nesprin during NET formation. In western blot analysis, we didn’t find protein bands with 11 kDa molecular weight as degradation products of Nesprin. Since the immunogen region of used anti-Nesprin-1 antibody does not cover the highly conserved spectrin domains.

### Calpain-1 mediated degradation of nesprin-1 collapses the nuclear scaffold

**Fig. 4:**
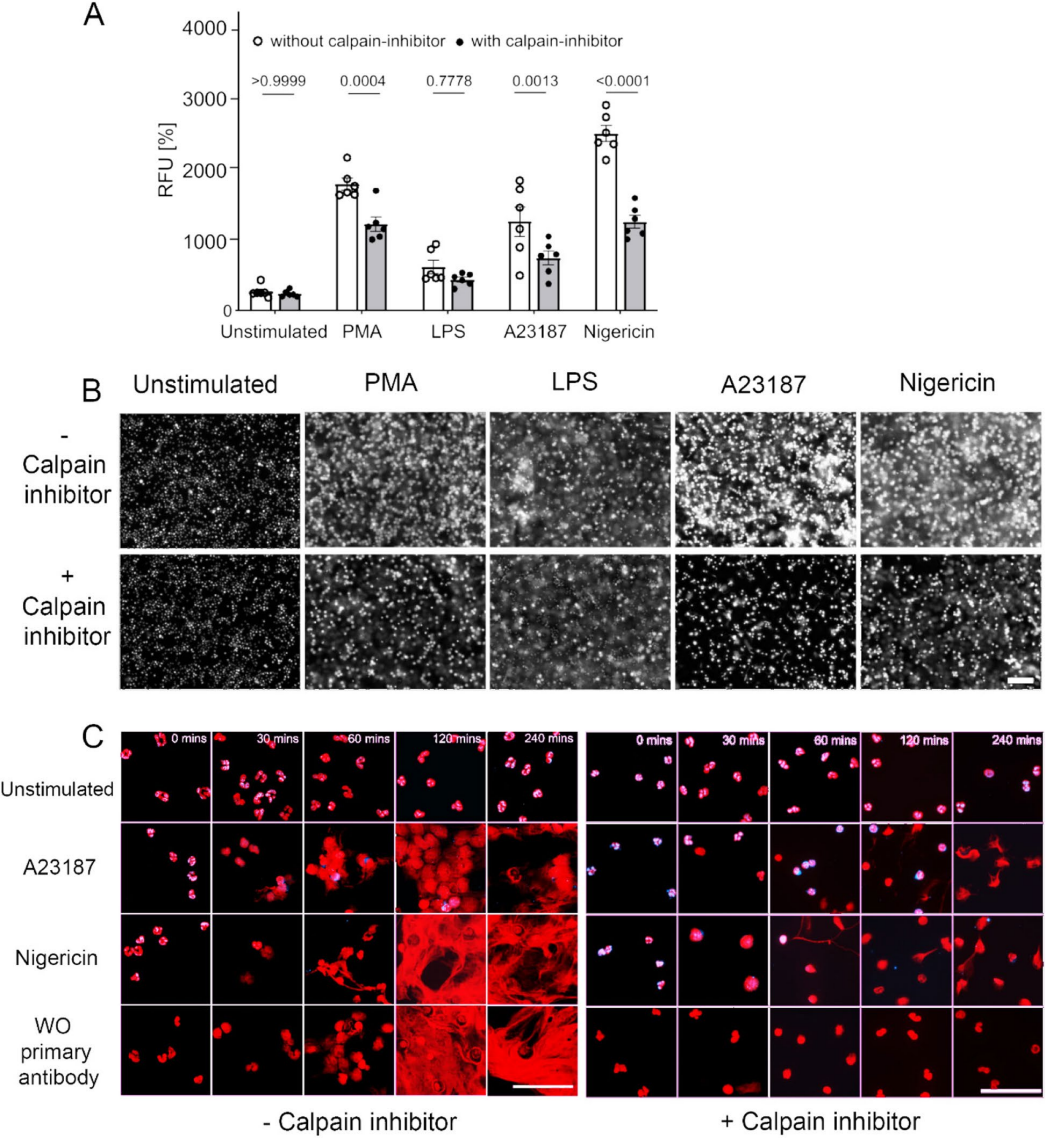
Calpain activation results in nesprin-1 degradation and nuclear envelope collapse. **A** Neutrophils were stimulated with PMA, LPS, A23187 and nigericin with (●) and without (○) 100 µM calpain-inhibitor calpeptin. Quantification of extranuclear chromatin showed profound inhibition of chromatin externalization (n = 6) in the presence of calpeptin. Data presented as mean ± SEM; two-way ANOVA and Bonferroni's multiple comparisons tests were used. **B** SYTOX Green stained DNA shows a decrease in chromatin externalization in the presence of calpeptin. Note, that especially the ionophore-driven chromatin externalization was affected. Scale bar 100 µm. **C** Microscopic image composites show inhibition of nesprin degradation in the presence of calpeptin during NET formation until 60 to 120 min compared to neutrophils stimulated in the absence of calpeptin. The scale bar is 50 µm

We then aimed to investigate the mechanism by which full-length nesprin is cleaved during NET formation. As previous study reported that PAD-4 activates calpain [20], we sought to examine the consequence of calpain-1 on nesprin during NET release. Neutrophils were incubated with NET inducers in the presence of extracellular Ca^2+^ with and without the cell-permeable calpain-inhibitor calpeptin. The latter inhibited chromatin externalization (Fig. 4A). Microscopic evaluation at 4 h confirmed the reduction of NET formation in the presence of a calpeptin (Fig. 4B). Furthermore, we monitored the appearance of nesprin-1 during the process of NET formation for over 4 h; to this end, we employed immunofluorescence imaging in the presence of calpeptin and label-free tomographic live cell imaging (Fig. 4C and Fig. S4A&4B). Immunofluorescence imaging revealed an inhibitory effect on nesprin-1 degradation and NET formation by calpeptin when the neutrophils had been stimulated with A23187 or nigericin (Fig. 4C).

Next, we took advantage of label-free live cell imaging of human blood neutrophils stimulated with A23187 with or without calpeptin. Neutrophils triggered with A23187 showed rapid loss of multi-lobulated nuclei and decondensed chromatin expanding in the cytoplasmic space accompanied by the plasma membrane shedding microvesicles (Fig. S4A movie). We observed less NET formation in the presence of calpeptin and the persistence of multiple lobes of the nuclei until the late phases of NET formation (Fig. S4B movie). We conclude that calpain-1 activation results in nesprin-1 shattering during the early phase of NET formation and prevents the nuclear envelope from protecting chromatin.

### Breakdown of nesprin-1 during A23187 induced NET formation is accompanied by sequential breakdowns of the LBR and actin cytoskeleton

**Fig. 5:**
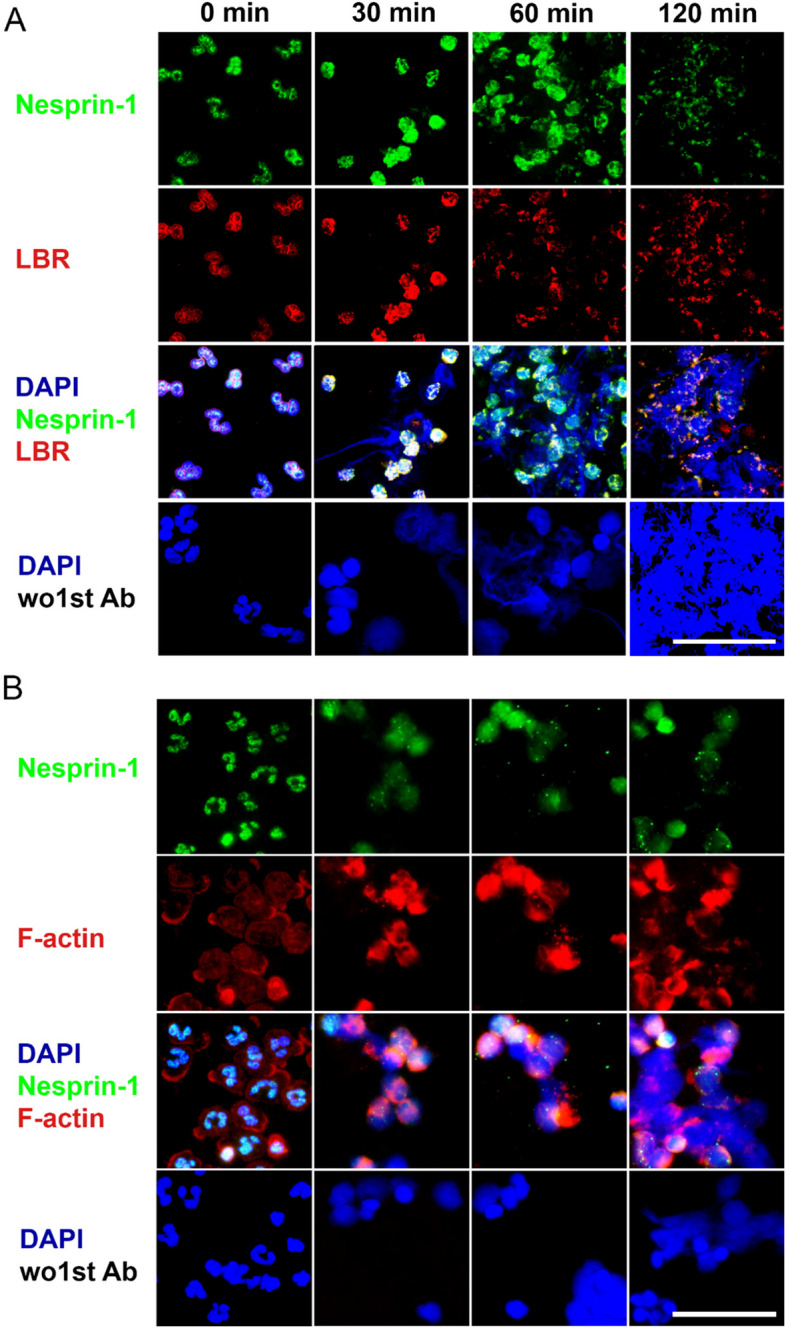
Lamin B receptor (LBR) and actin are rearranged during calcium ionophore-induced NETs formation. Immunofluorescent staining exhibits the disassembly of the inner nuclear membrane simultaneously and the actin cytoskeleton in a later period, as nesprin-1 proceeds to break over two hours following incubation of neutrophils with calcium ionophore A23187. **A** Nesprin-1 and LBR were seen along the rims of the multilobulated nuclei, followed by rounding of the cores and weak nesprin-1 and LBR fluorescence as neutrophils proceeded to NETs (**B**) the actin cytoskeleton shows only a faint staining in healthy neutrophils. The bright actin labelling along the nucleus appeared mostly undisrupted in the early phase of NET formation. Fluorescence microscopy analyses for nesprin-1 (green), inner nuclear membrane protein lamin B receptor (LBR) (red) (**A**) and actin cytoskeleton (SiR-actin) (**B**), nuclear chromatin as stained for DAPI (blue). The scale bar is 50 µm

Next, we investigated the alterations of the nuclear membrane and the cytoskeleton in the course of NET formation. To this end, we stained for lamin B receptor (LBR) and actin cytoskeleton. Neutrophils incubated with A23187 (Fig. 5), nigericin (Fig. S5), or PMA (Fig. S6) showed the degradation of nesprin-1, LBR, and actin in a time dependent manner. Nesprin-1 broke down between 30 and 60 min, along with LBR disintegration after 120 min. There were only remnants of nesprin-1 and LBR to be seen residing on the NETs (Fig. 5A). The actin cytoskeleton persisted as nesprin-1 began to disassemble. Some of the actin could still be observed in cells that were already rounded (Fig. 5B).

We also investigated the nesprin-1, LBR and actin breakdown in neutrophils stimulated with nigericin and PMA. Similar to A23187, in nigericin-stimulated neutrophils nesprin-1 degradation and LBR disintegration occurred simultaneously and actin persisted for 120 min (Fig. S5A & 5B). PMA-stimulated neutrophils began to round up by 120 min, the nesprin-1 began to weaken, but LBR was still visible around the nucleus. After 240 min, NETs formed and nesprin-1 had mostly deteriorated, but much of the LBR fragments continue to linger (Fig. S6A). When NET formation reached its peak, the actin cytoskeleton was also fragmented and disappeared (Fig. S6B).

Taken together, we show that nesprin-1 and LBR breakdown go hand-in-hand when stimulated with ionophores such as A23187 and nigericin. Induction of neutrophils with PMA shows nesprin-1 to reorganize before LBR. The actin cytoskeleton remains visible for 120 min, despite the disappearance of nesprin-1, and LBR; thereafter, it also crumbles into pieces.

## Discussion

Even though many pathways involved in the formation of NET have been identified, the molecular mechanisms that drive the structural changes of nuclear envelope breakdown remain elusive. Nuclei in mature neutrophils possess a multilobulated structure. As neutrophils proceed towards NET formation, the nuclei begin to round up and distort. We investigated the role of Ca^2+^ dependent protease calpain in nuclear distortion during NET formation. We observed calpain mediated proteolysis of nesprin-1 in the LINC complex. The collapse of the nesprin framework reduces nuclear envelope crosslinking, and initiates disintegration of LBR and finally of the actin cytoskeleton.

Our data supports that the influx of Ca^2+^ choreographs the sequential increase of cytoplasmic and nuclear Ca^2+^, activates PAD-4, then calpain, and finally enables the release of NETs [20, 24]. Studies have shown that intracellular Ca^2+^ can, to some extent, further contribute to the increase of cytoplasmic Ca^2+^ by emptying intracellular Ca^2+^ stores such as ER or granules [25]; other studies show that chelation of extracellular Ca^2+^ inhibits A23187-induced NET formation. This shows that Ca^2+^ of the extracellular environment is required for the formation of NETs [26–28]. Our data confirm that an influx of extracellular Ca^2+^ is necessary to instigate NETs; chelation of extracellular Ca^2+^ reduced formation of NETs.

The giant isoform from the family of nesprin proteins, nesprin-1, bridges the actin cytoskeleton to the nuclei and, thus, organizes nuclear localization [29]. Our data show that the nesprin-1 of neutrophil stabilizes the nuclear membrane and safeguards the chromatin from externalization. The disruption of nesprin-1 causes the nucleus to lose its lobulated structure and swell; leaving behind the dot-like remains of the disrupted nesprin-1 meshwork. The disrupted nuclear envelope allows chromatin externalization. In short, nesprin-1 degradation during NET formation results in loss of nuclear membrane morphology. This suggests that nesprin-1 plays a vital role in the maintenance and morphology of the nuclei of neutrophils. This is in line with in vivo analyses of nesprin-1 knockout mice with altered nuclear morphology, nuclear envelope organization, actin organization, and cell motility [29–33]. Nesprin-1 knockdown cell lines displayed mislocalization and nuclear defects in human osteosarcoma U2OS cell lines and fibroblasts [34]. Furthermore, our co-immunofluorescence data of nesprin-1 and DNA revealed a decreased ratio of Nesprin/DAPI in the course of NET formation. Nesprin-1 degradation resulted in chromatin externalization. Thus, our results are consistent with the knockout models from in vivo and in vitro studies with different types of cells, showing that the breakdown of nesprin-1 adversely affects the nuclear morphology and chromatin expulsion in neutrophils, which, to our knowledge, has not been reported before.

The presence of a filamentous nesprin-based meshwork, provides a structural support to outer nuclear membrane and links the outer nuclear surface to its cellular surrounding [35]. It has been reported that the apoptotic stimuli can induce degradation and subcellular redistribution of nesprin-1 and nesprin-2 in neutrophils [36]. In line with this report we found that the nesprin from LINC complex of nuclear envelope is targeted and this resulted in the loss of nuclear integrity before NET release.

The molecular weight of the giant nesprin-1 isoform has been calculated to approx. ~ 800 to 1000 kDa and comprises 74 spectrin repeat (SR) domains which equip the nesprin-1 protein to connect the cell's nuclear envelope to the F-actin via the N-terminal calponin homology domains [10, 37]. Our data suggests that stimulating neutrophils with various inducers results in shorter nesprin-1 fragments (9 or 11 kDa), allowing the nuclei of the neutrophils to disintegrate and release NETs. Such a result indicates a proteolytic enzyme cleaves between the SR domains comprising 87% of the entire length of nesprin-1. Interestingly, Ca^2+^-dependent cytosolic cysteine protease calpain has been known to proteolyze several membrane-bound and membrane-associated proteins as a substrate, including spectrin [14, 38]. In addition, calpain has been shown to produce spectrin breakdown products and cleave the inter domain linkers of αII-Spectrin and β-Spectrin [14, 39–41]. Consistent with our hypothesis, calpain activation has been shown to specifically break αII-Spectrin, a neuronal cytoskeleton protein, in various in vitro and in vivo neuronal injury models [42]. Also, active calpain reportedly degraded several nucleoporins and caused concomitant loss of nucleo-cytoplasmic portioning of reporter molecules in primary cortical neurons [43]. These studies indicate that calpain is responsible for the breakdown of spectrin repeat domains of full-length nesprin-1, leaving behind small-length fragments corresponding to the size of an individual spectrin repeat domain. Here, we enlisted the cleavage sites of Nesprin-1 protein by calpain using computational tool named DeepCalpain (http://deepcalpain.cancerbio.info/) (Table S1) [44]. But, future studies are required to investigate the calpain mediated proteolytic cleavage mechanism of Nesprin-1 in detail.

An increase of the Ca^2+^ concentration in the cytosol of various cell systems has been shown to regulate calpain activation. [45, 46]. We have confirmed that a concerted contribution of Ca^2+^, PAD-4 and calpain increase the nuclear swelling and chromatin decondensation. Indeed, we observed an inhibition of NET formation by the calpain inhibitor calpeptin; especially in the presence of A23187 or nigericin. Stimulating neutrophils with PMA induces NET formation independent of extracellular Ca^2+^. However, it displayed decreased NET formation when calpain was inhibited. Similar observations have been reported for calpain–mediated X-linked inhibitor of apoptosis (XIAP), where calpain-mediated degradation of XIAP led to the initiation of apoptosis in normal neutrophils and dysfunction of this regulatory pathway that resulted in pathological accumulation of neutrophils [47]. When calpain activity was inhibited using calpeptin in neutrophils stimulated with the calcium ionophore A23187, XIAP-cleavage was abolished in a time-dependent manner, further emphasizing the role of calpain mediated proteolysis in neutrophils [47].

Nuclear envelope breakdown and externalization of decondensed chromatin are the hallmarks of the process of NET formation. The vesicular budding of nuclear envelope during NET formation has been evidenced by transmission electron microscopy (TEM) of in vitro stimulated neutrophils. Immunostainings of PMA stimulated neutrophils detected LBR in nuclear membrane vesicles [1]. Impaired LBR expression in neutrophils has been previously reported to cause Pelger-Huet anomaly. This disease shows alteration of the neutrophil nuclear morphology [48, 49], promoting lupus autoimmunity in a mouse model [50]. In line with this we observed the disintegration of LBR simultaneously with nesprin-1 in A23187 or nigericin induced NET formation. PMA-triggered NETs showed nesprin-1 disintegration is followed by LBR disintegration. The nuclear membrane protein nesprin-1 on the ONM and LBR on the INM are targeted during the initial stages of NET formation. This results in nuclear envelope rupture.

We then investigated the consequence of nesprin-1 breakdown on the interacting F-actin cytoskeleton. The degradation of actin cytoskeleton has been observed in neutrophils when activated with *Candida albicans*. It has been demonstrated to be essential for NET formation [51, 52]. We found that nesprin-1 degraded rapidly in the early phase of treatment with nigericin and A23187; however, the actin cytoskeleton was preserved up to 60 and 120 min, respectively. Similarly, as nesprin-1 degradation starts 120 min after PMA challenge, the actin pieces as NET formation was completed. Our findings are supported by previous studies reporting that a functional actin cytoskeleton still operates in the early stages of NET formation [53].

There are two types of NET formation: vital and non-vital [54]. In non-vital NET formation, DNA release is associated with the cell death. Here, we have studied mainly in vitro non-vital NET formation. We propose that active calpain degrades proteins of the nuclear membrane like nesprin-1. This causes nuclear breakdown and is followed by the lytic phenomenon. While, in vital NET formation, neutrophil DNA is released through nuclear envelop budding and vesicular formation. The cell body remains intact. We speculate that calpain activity is still involved in nuclear breakdown and DNA release. However, the lytic phenomenon is prevented because the cells remain mobile and controlled by additional signals mediated by cell receptors and cytoplasmic molecules. Future studies are required to investigate this concept.

Taken together we propose that as extracellular Ca^2+^ enters the neutrophil, ROS are generated; as a consequence, PAD-4 is activated and triggers calpain to cleave the nesprin-1 on ONM. This initiates the disintegration of the nuclear envelope, damages LBR lodged in the INM and results in the cytoplasmic appearance of decondensed chromatin. The actin cytoskeleton still persists in the early phases of NET formation, while other components, especially nesprin-1, is degraded. This can be attributed to the actin-dependent positioning of the nucleus in the vicinity of the plasma membrane before the disintegration of the nuclear envelope and finally NET release [55].

## Supplementary Information

Supplementary Material 1.

## Data Availability

No datasets were generated or analysed during the current study.
