# Supplementary material for: Calpain-1 weakens the nuclear envelope and promotes the release of neutrophil extracellular traps
Source: Cell Commun Signal. 2024 Sep 9;22:435. doi: 10.1186/s12964-024-01785-6 (PMC11384698; doi:10.1186/s12964-024-01785-6)
Supplement: Supplementary file 1 — Supplementary Material 1. [file 12964_2024_1785_MOESM1_ESM.zip › 2024-08 4d99e6df-ce57-40e4-b8f8-66d6397f92fc Supplementary data.docx]

**Supplementary data**


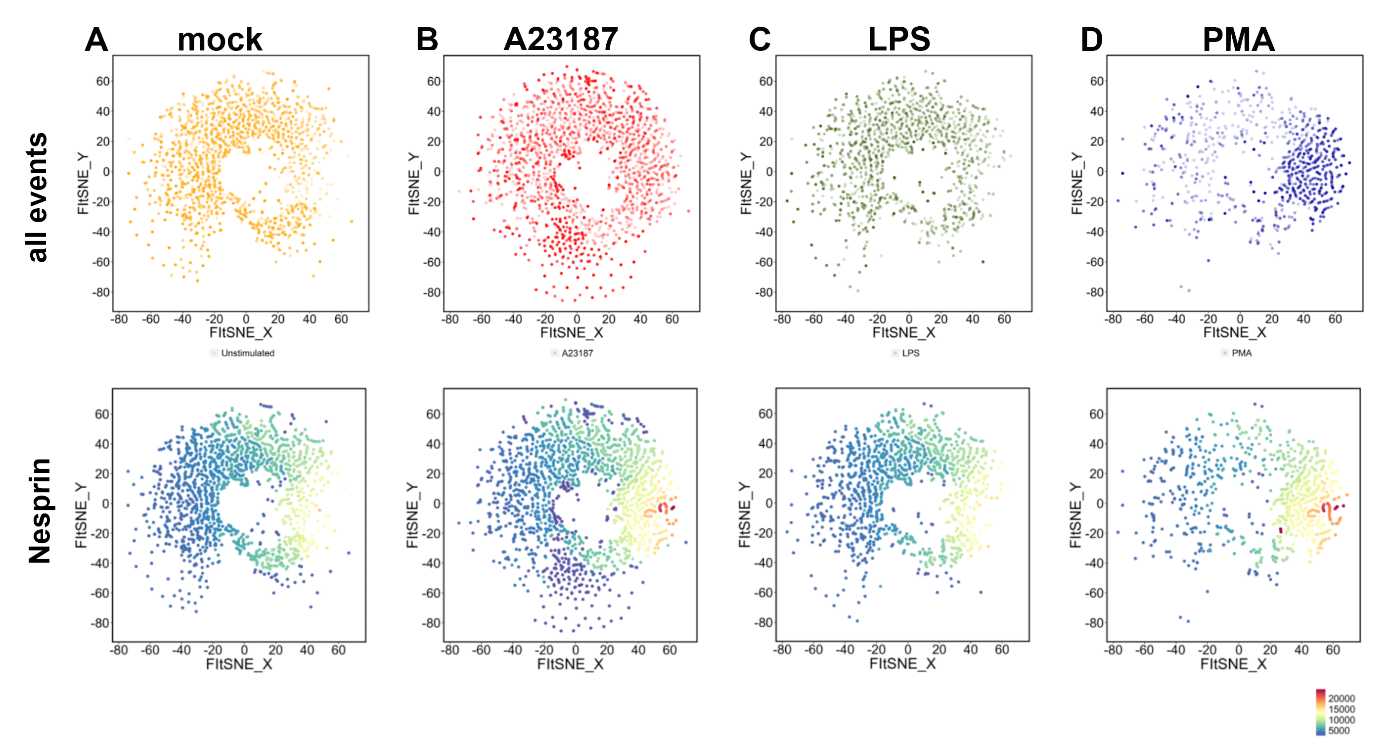


**Fig S1 The fluorescence pattern of Nesprin-1 depends on the NET inducer.**

FIt-SNE plots of unstimulated neutrophils, calcium ionophore A23187, LPS, and PMA stimulated neutrophils displaying morphometric clusters (upper panels). The signal intensity of nesprin-1 is displayed in the lower panels. Note, the population with lowest nesprin-1 fluorescence colored in violet appears mostly for A23187 stimulated neutrophils.

**
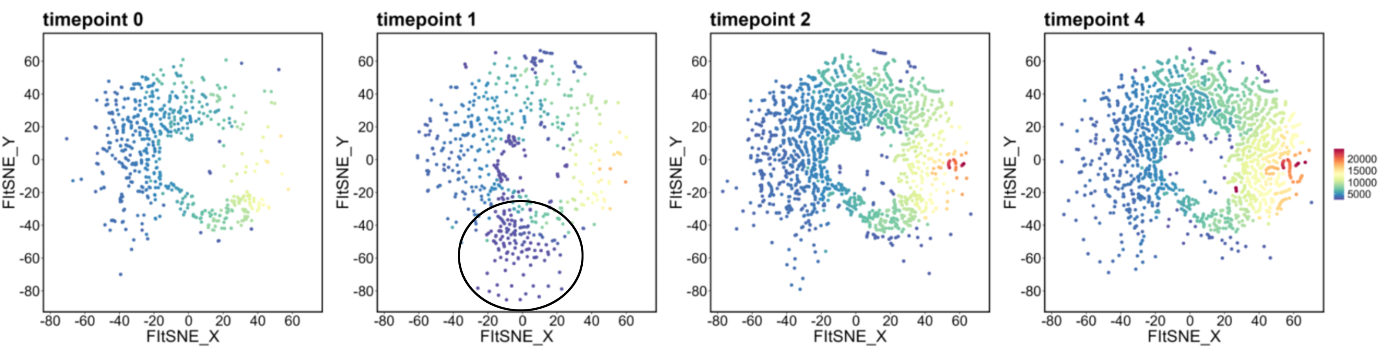
**

**Fig S2 During the course of NET formation the Nesprin-1 fluorescence pattern changes with time**

FIt-SNE plots of ionophore A23187-stimulated neutrophils showing a morphometric cluster with very low nesprin-1 expression only after one hour of incubation.


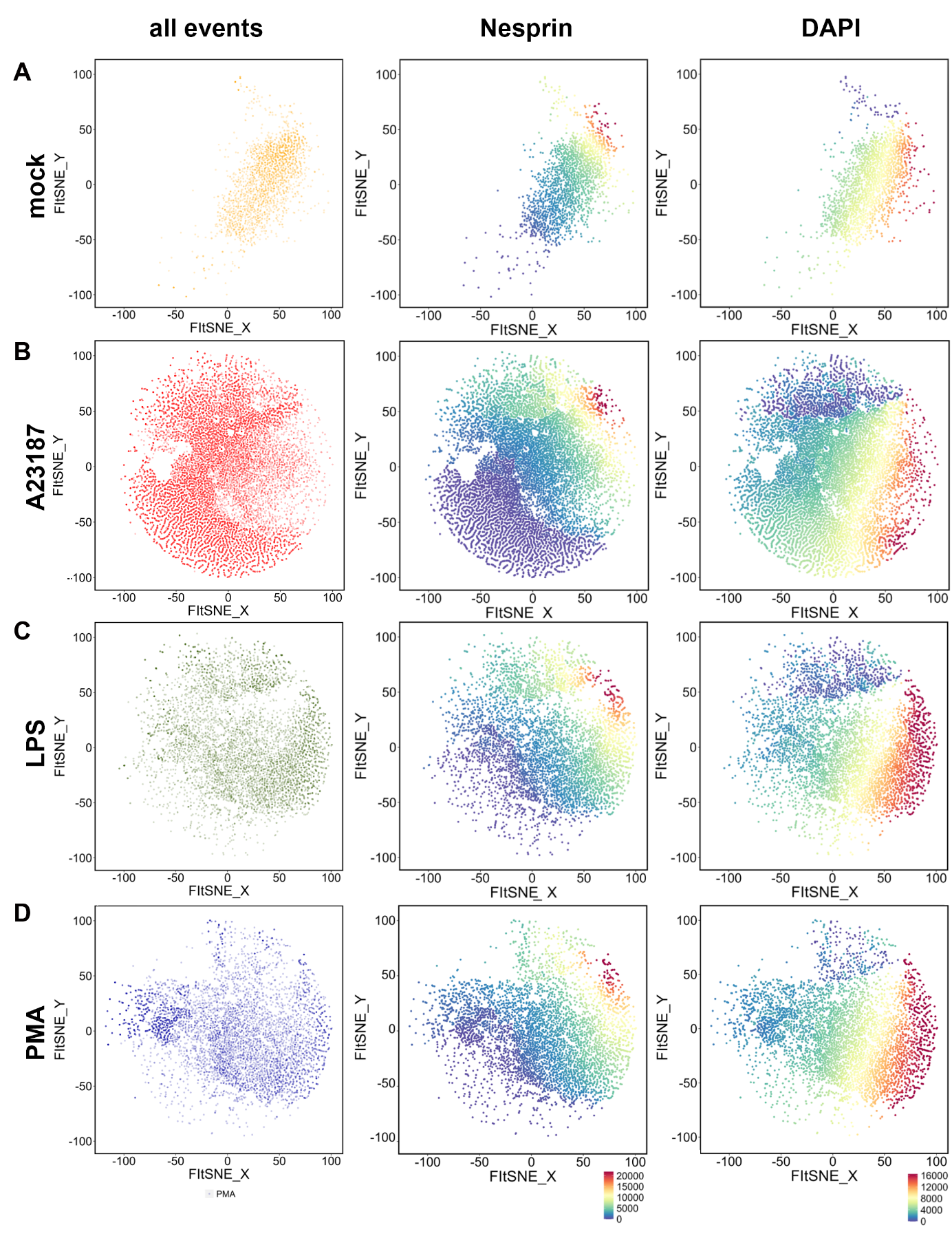


**Fig S3 NETs induced by different NET inducers can be distinguished by Nesprin and DAPI pattern.**

FIt-SNE plots of (A) unstimulated (B) stimulated with calcium ionophore A23187 (C) LPS and (D) PMA NETs showing morphometric clusters (left panels). The fluorescence intensity of Nesprin-1 and DAPI are indicates in the right panels. Note that the different inducers have unique Nesprin-DAPI pattern.

**Figure S4A Video Neutrophils stimulated with A23187 calcium ionophore loose lobular nuclear morphology and showed increase in expansion of cytoplasmic space.** Neutrophils were cultured in a 35 µm plate with a glass bottom incubated with NET inducer A23187 at 37˚C and was continuously monitored for 4 hours. Images by Nanolive 3D Cell Explorer®

**Figure S4B Video Calpeptin treated neutrophils stimulated with A23187 calcium ionophore maintain the nuclear lobulated structure and bypass NET formation**. Neutrophils incubated with 100µM calpeptin and stimulated with calcium ionophore A23187 at 37˚C and were continually observed for 4 hours. Imaged by Nanolive 3D Cell Explorer®.


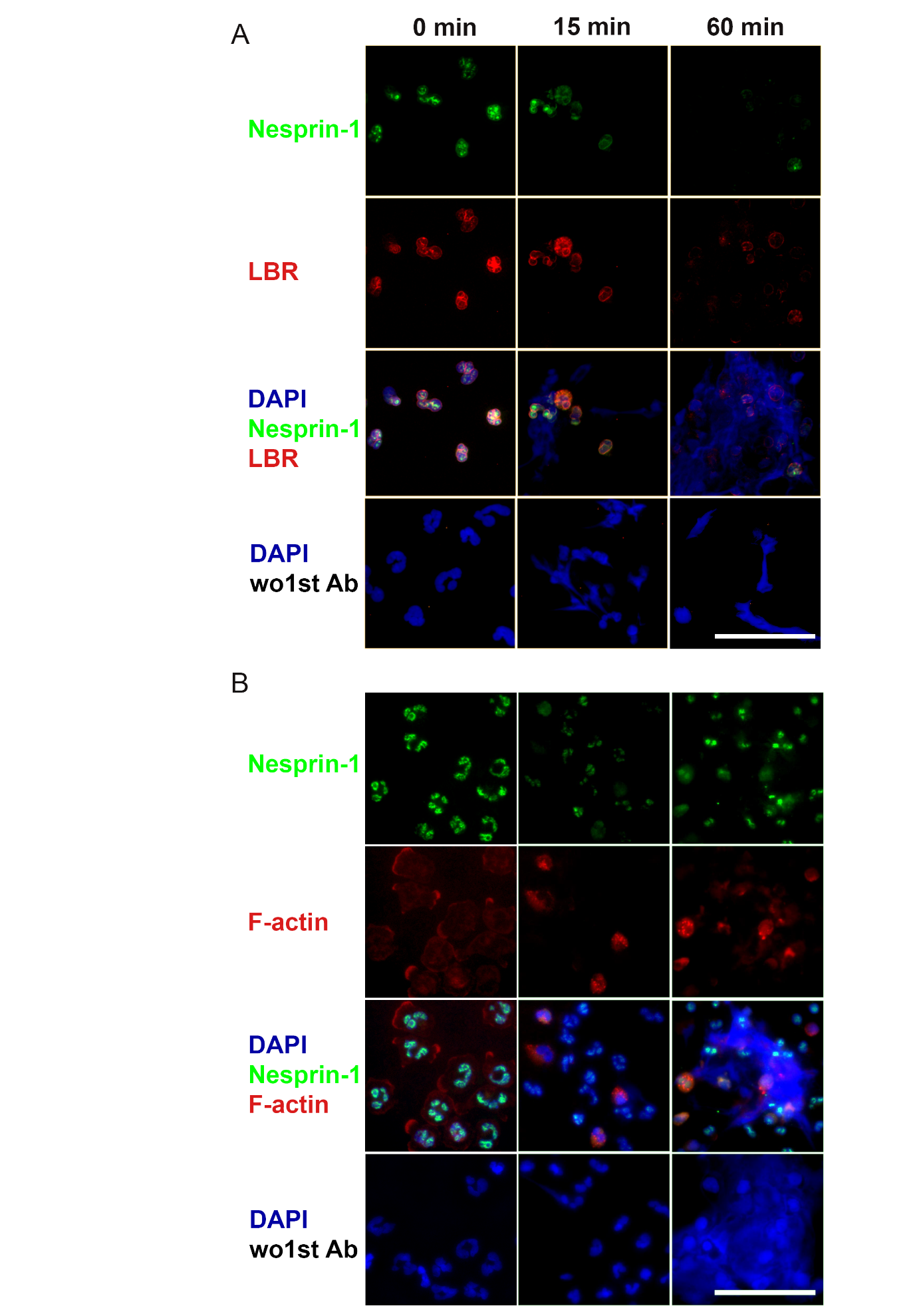


**Figure S5 Nesprin-1, LBR, and F-actin are consecutively degraded during nigericin-induced NET formation**

(A) Nesprin-1 deterioration in healthy isolated neutrophils stimulated with nigericin lose the inner nuclear membrane protein LBR as NETs proceed to form however (B) some actin cytoskeleton remains preserved. The scale bar is 50µm. Note that Nesprin-1 and LBR are degraded simultaneously. Note, that nesprin-1 and LBR degradation precede the resolution of the F-actin cytoskeleton.


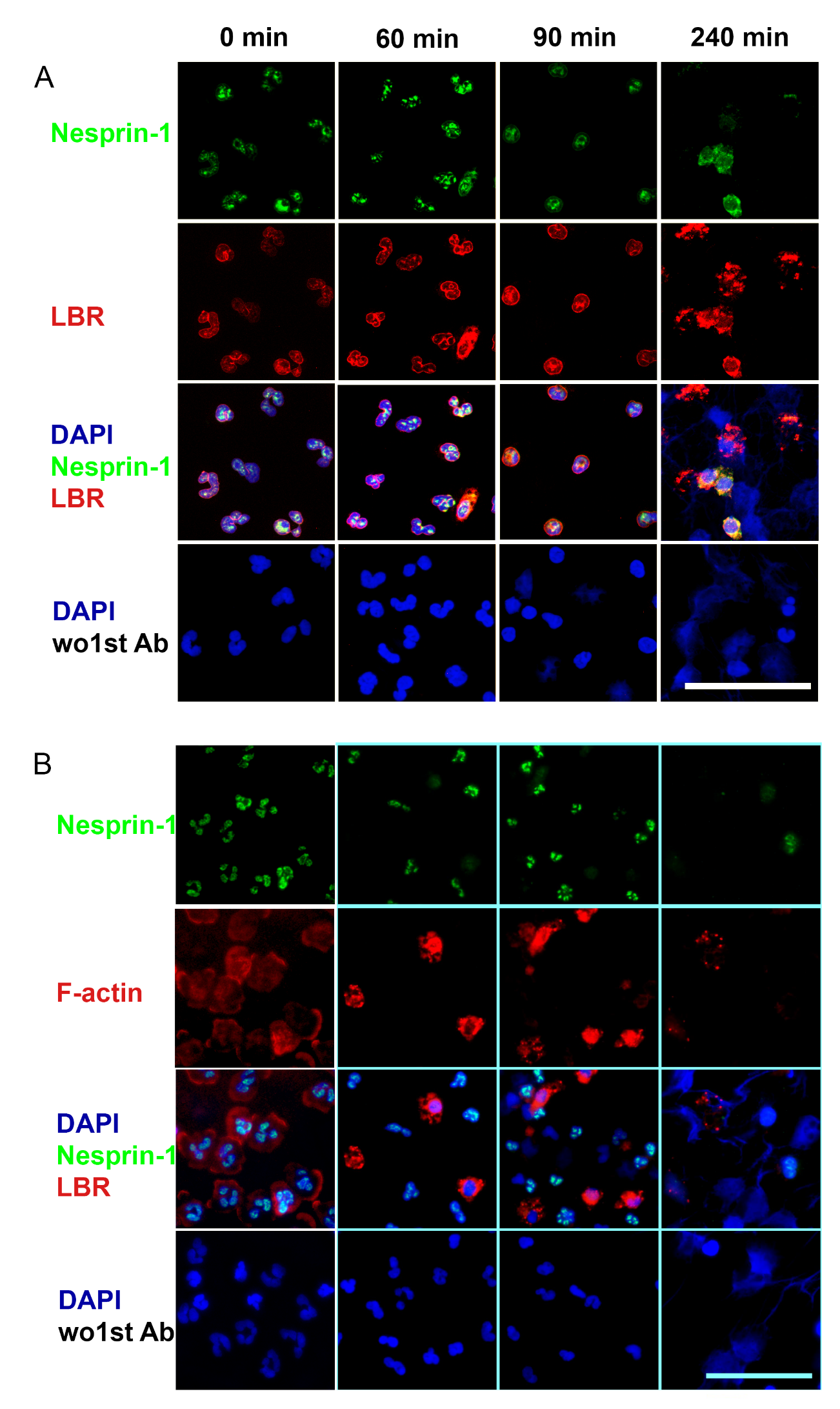


**Figure S6 Nesprin-1, LBR, and F-actin are consecutively degraded during PMA-induced NET formation**

1. LBR staining of PMA stimulated neutrophils to show LBR preserved around the rims of the lobulated nuclei by 60min time-point and beginning to round and continue to persist and deteriorate by 120 min and 240 min respectively, as nesprin-1 disintegrates. (B) Actin cytoskeleton erupts and fragments by 240 min. The scale bar is 50µm.Note that nesprin-1 and LBR are degraded consecutively in PMA-induced NETs.


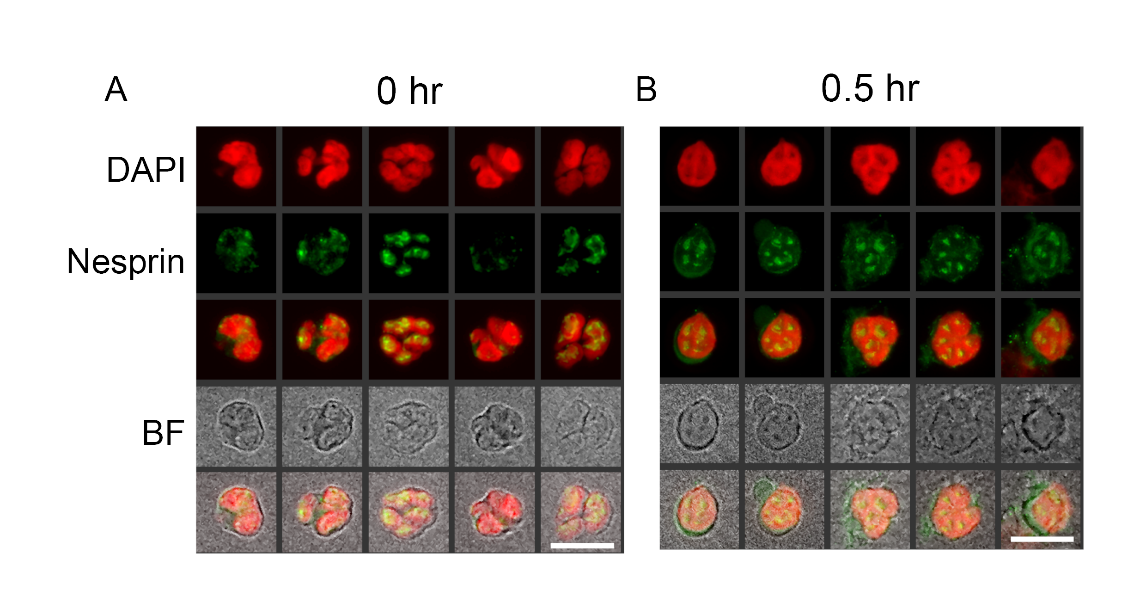


Figure S7 Immunofluorescence staining of neutrophils for DNA (DAPI displayed in red) and nesprin-1 (displayed in green) before and 30 minutes after stimulation with calcium ionophore A23187 (1mM Ca^2+^). Merge composites, nesprin-1 (green), DNA (red) and bright field (BF) images demonstrates the breakdown of nesprin-1 from neutrophil nuclear membrane within 30 minutes. It leaves dot-like nesprin-1 remnants, and is accompanied by the loss of nuclear lobes. These nesprin-1 remnants are within the cell. Scale bar is 20 µm.

Table 1 Cleavage sites in Nesprin-1 by calpain can be predicated bioinformatically. The following predicted sites of cleavage were listed using online computational tool http://deepcalpain.cancerbio.info.

|  |  |  |  |
| --- | --- | --- | --- |
| Position | Enzyme | FPR | Peptide |
|  |  |  |  |
| 1335 \| 1336 | calpain | 0,58% | RTESIAVQ\|AENLVKEA |
| 1825 \| 1826 | calpain | 0,97% | ASLSGILR\|QLRQTVEA |
| 1920 \| 1921 | calpain | 0,81% | ADALAVLK\|KAFQDQKE |
| 2841 \| 2842 | calpain | 0,53% | ASRLSRVE\|SLAPEVKQ |
| 3198 \| 3199 | calpain | 0,20% | RHRVSQLS\|SQYLALSN |
| 3283 \| 3284 | calpain | 0,12% | QMKMIVTR\|GESVLQNT |
| 3410 \| 3411 | calpain | 0,45% | QLELQDLQ\|ERYRAIQE |
| 3413 \| 3414 | calpain | 0,29% | LQDLQERY\|RAIQERAK |
| 3418 \| 3419 | calpain | 0,78% | ERYRAIQE\|RAKEAVTK |
| 3570 \| 3571 | calpain | 0,98% | PRTRRQSN\|RATKEIQL |
| 3571 \| 3572 | calpain | 0,23% | RTRRQSNR\|ATKEIQLH |
| 3579 \| 3580 | calpain | 0,73% | ATKEIQLH\|QMKKWHEE |
| 3727 \| 3728 | calpain | 0,68% | EGEAERLR\|KEIHDHME |
| 3738 \| 3739 | calpain | 0,94% | HDHMEQLK\|ELTSTVRK |
| 5668 \| 5669 | calpain | 0,79% | RLQHTAIQ\|QCNIMQEA |
| 5715 \| 5716 | calpain | 0,29% | TSNIQELQ\|AQISRHEE |
| 5843 \| 5844 | calpain | 0,82% | IAYYQALS\|AERLQTDA |
| 6211 \| 6212 | calpain | 0,74% | AAETSGDA\|GEKPDVLS |
| 6223 \| 6224 | calpain | 0,49% | DVLSQELG\|MEGEKSSA |
| 6588 \| 6589 | calpain | 0,86% | MILTETLF\|RKIISFAV |
| 6597 \| 6598 | calpain | 0,41% | KIISFAVQ\|KETQFHTE |
| 6599 \| 6600 | calpain | 0,92% | ISFAVQKE\|TQFHTELM |
| 6603 \| 6604 | calpain | 0,99% | VQKETQFH\|TELMAQAS |
| 6607 \| 6608 | calpain | 0,58% | TQFHTELM\|AQASAVLK |
| 6649 \| 6650 | calpain | 0,29% | SRQLEVVE\|SSIPSVGL |
| 6650 \| 6651 | calpain | 0,45% | RQLEVVES\|SIPSVGLV |
| 6801 \| 6802 | calpain | 0,27% | SLKSSVLS\|TGNQLLRL |
| 6808 \| 6809 | calpain | 0,25% | STGNQLLR\|LKKVDTAT |
| 6884 \| 6885 | calpain | 0,76% | IKNSIGYK\|AIHEYLQK |
| 7637 \| 7638 | calpain | 0,30% | RTFKKKLS\|QSLPDHHE |
| 7638 \| 7639 | calpain | 0,35% | TFKKKLSQ\|SLPDHHEE |
| 772 \| 773 | calpain | 0,67% | TSFYDSLG\|KINEIITV |
| 8198 \| 8199 | calpain | 0,53% | PQPSSNLS\|LSLAQPLR |
| 8200 \| 8201 | calpain | 0,54% | PSSNLSLS\|LAQPLRSE |
| 8201 \| 8202 | calpain | 0,90% | SSNLSLSL\|AQPLRSER |
| 8206 \| 8207 | calpain | 0,23% | LSLAQPLR\|SERSGRDT |
| 8298 \| 8299 | calpain | 0,98% | QTENIIRS\|KTPTGPEL |
| 8365 \| 8366 | calpain | 0,33% | IDRWELLQ\|AQALSKEL |
| 993 \| 994 | calpain | 0,29% | TKLMPQEG\|SEKIIKEH |
|  |  |  |  |
| 104 \| 105 | m calpain | 0,33% | SIVSSETP\|SPPSKRKV |
| 114 \| 115 | m calpain | 0,82% | PSKRKVTT\|KIQGNAKK |
| 3283 \| 3284 | m calpain | 0,45% | QMKMIVTR\|GESVLQNT |
| 3410 \| 3411 | m calpain | 0,30% | QLELQDLQ\|ERYRAIQE |
| 3425 \| 3426 | m calpain | 0,69% | ERAKEAVT\|KSEKLVRL |
| 3426 \| 3427 | m calpain | 0,40% | RAKEAVTK\|SEKLVRLH |
| 3579 \| 3580 | m calpain | 0,70% | ATKEIQLH\|QMKKWHEE |
| 3738 \| 3739 | m calpain | 0,47% | HDHMEQLK\|ELTSTVRK |
| 5715 \| 5716 | m calpain | 0,92% | TSNIQELQ\|AQISRHEE |
| 5772 \| 5773 | m calpain | 0,49% | AEGTEDLD\|GELLPTPS |
| 5776 \| 5777 | m calpain | 0,86% | EDLDGELL\|PTPSAHPS |
| 5779 \| 5780 | m calpain | 0,93% | DGELLPTP\|SAHPSVVM |
| 8298 \| 8299 | m calpain | 0,55% | QTENIIRS\|KTPTGPEL |
| 8645 \| 8646 | m calpain | 0,64% | TPRGKCSL\|SQPGPSVS |
| 8646 \| 8647 | m calpain | 0,85% | PRGKCSLS\|QPGPSVSS |
| 8732 \| 8733 | m calpain | 0,40% | HPMLRYTN\|GPPPL--- |
|  |  |  |  |
| 2160 \| 2161 | u calpain | 0,73% | SNNCVPQM\|AENISNLD |
| 2316 \| 2317 | u calpain | 0,48% | KYHSAELE\|SLGRAMTG |
| 2319 \| 2320 | u calpain | 0,62% | SAELESLG\|RAMTGLIK |
| 2960 \| 2961 | u calpain | 0,75% | EPRTEDLK\|SQLNELCR |
| 3198 \| 3199 | u calpain | 0,38% | RHRVSQLS\|SQYLALSN |
| 4177 \| 4178 | u calpain | 0,74% | LQREEDLQ\|RTRDYHDC |
| 5234 \| 5235 | u calpain | 0,72% | PGEEPPLM\|QEITAMQD |
| 5656 \| 5657 | u calpain | 0,76% | CHAALRLQ\|EEASRLQH |
| 5843 \| 5844 | u calpain | 0,30% | IAYYQALS\|AERLQTDA |
| 5961 \| 5962 | u calpain | 0,97% | QDEINELQ\|SSLAEELV |
| 5969 \| 5970 | u calpain | 0,98% | SSLAEELV\|SESCEADP |
| 6145 \| 6146 | u calpain | 0,34% | EESDVDLT\|ATQSPGVQ |
| 6148 \| 6149 | u calpain | 0,58% | DVDLTATQ\|SPGVQEWL |
| 6790 \| 6791 | u calpain | 0,87% | LEFSKEVD\|AQSSLKSS |
| 6793 \| 6794 | u calpain | 0,97% | SKEVDAQS\|SLKSSVLS |
| 6796 \| 6797 | u calpain | 0,61% | VDAQSSLK\|SSVLSTGN |
| 6799 \| 6800 | u calpain | 0,71% | QSSLKSSV\|LSTGNQLL |
| 6800 \| 6801 | u calpain | 0,53% | SSLKSSVL\|STGNQLLR |
| 6801 \| 6802 | u calpain | 0,29% | SLKSSVLS\|TGNQLLRL |
| 7206 \| 7207 | u calpain | 0,75% | EDRTNELL\|KAATNKDI |
| 7207 \| 7208 | u calpain | 0,40% | DRTNELLK\|AATNKDIA |
| 8019 \| 8020 | u calpain | 0,35% | TDSACSLK\|QMVHEGNQ |
| 8189 \| 8190 | u calpain | 0,50% | DRSADSLL\|SPQPSSNL |
| 8190 \| 8191 | u calpain | 0,25% | RSADSLLS\|PQPSSNLS |
| 8193 \| 8194 | u calpain | 0,37% | DSLLSPQP\|SSNLSLSL |
| 8194 \| 8195 | u calpain | 0,64% | SLLSPQPS\|SNLSLSLA |
| 8195 \| 8196 | u calpain | 0,85% | LLSPQPSS\|NLSLSLAQ |
| 8196 \| 8197 | u calpain | 0,28% | LSPQPSSN\|LSLSLAQP |
| 8197 \| 8198 | u calpain | 0,46% | SPQPSSNL\|SLSLAQPL |
| 8198 \| 8199 | u calpain | 0,06% | PQPSSNLS\|LSLAQPLR |
| 8199 \| 8200 | u calpain | 0,16% | QPSSNLSL\|SLAQPLRS |
| 8200 \| 8201 | u calpain | 0,13% | PSSNLSLS\|LAQPLRSE |
| 8201 \| 8202 | u calpain | 0,13% | SSNLSLSL\|AQPLRSER |
| 8202 \| 8203 | u calpain | 0,47% | SNLSLSLA\|QPLRSERS |
| 8206 \| 8207 | u calpain | 0,22% | LSLAQPLR\|SERSGRDT |
| 8232 \| 8233 | u calpain | 0,66% | WDHDYDLS\|RDLESAMS |
| 8236 \| 8237 | u calpain | 0,25% | YDLSRDLE\|SAMSRALP |
| 8365 \| 8366 | u calpain | 0,43% | IDRWELLQ\|AQALSKEL |
| 8378 \| 8379 | u calpain | 0,68% | KELRMKQN\|LQKWQQFN |
| 8380 \| 8381 | u calpain | 0,21% | LRMKQNLQ\|KWQQFNSD |
| 8630 \| 8631 | u calpain | 0,94% | SVSPTSGR\|STPNRQKT |
